# Supplementary material for: Evaluating source credibility effects in health labelling using vending machines in a hospital setting
Source: PLoS One. 2024 Feb 16;19(2):e0296901. doi: 10.1371/journal.pone.0296901 (PMC10871505; doi:10.1371/journal.pone.0296901)

Supplementary Information: Methods

*Implementation of study*

Vending machines were in public areas, where staff, patients and visitors could all access the machines. One machine was in an Accident and Emergency department (*A&E* machine), the other in a reception area (*reception* machine). The A&E machine was in an isolated location, separated from other food outlets. The reception machine was located within the main entrance for maternity patients, and on the same floor as a coffee shop and restaurant, which sold a variety of snacks and hot and cold beverages.

Vending machines were identical (Model: Necta Tango) running the Nayax vending system. The machines allowed cash and card purchases. They had 32 coils, 16 for crisps (four products in each of the four top rows) and 16 for bars/small packets (eight products in each of the bottom two rows). Healthy products were given the most prominent locations within this set up (top two crisp rows, top confectionary row) to maximize their visibility within the machines.  Product sold, location, exact time, and method of purchase (cash or card; first and last four digits recorded if card) were all recorded by the Nayax system.

Planograms (diagrams of the planned visual layout of stock) were kept constant throughout the trial (see SI.1). The fidelity of the compliance to this layout was monitored at each labelling condition changeover. Time logs of each changeover period during the trial were recorded, to ensure consistency and to allow cross-referencing with the sales data to ensure all sales were logged under the correct condition.

## *Vending snacks*

Snacks were selected by the vending provider (JDJ Vending Services) and the research team (including a Public Health Wales Consultant and a Public Health Wales Dietitian). All snacks were selected with the aim of maximizing profit subject to the healthy/unhealthy experimental constraints.

Healthy snacks satisfied the Welsh Hospital Healthy Vending directive constraints (Welsh Government, 2012) whereas unhealthy products did not. For the healthy condition, there were 12 distinct snacks, 4 of which occupied two coils, and for the unhealthy condition, there were 11 distinct snacks, 5 of which occupied two coils. Details of the nutritional information for each individual snack can be seen in SI.2. The mean wholesale cost of healthy products was greater than that of the unhealthy products (Healthy = 54p (SD = 7p); Unhealthy = 43p (SD = 5p)), and all products were sold for £1.

## *Labels*

The labels were placed underneath the product it referred to, after its coil number (see SI.3). Labels were 48mm wide and 18mm high and were vinyl removable adhesives. This allowed for clean removal of labels during each changeover period.

The lighter choices claim was designed as a variation of the “NHS Choices” logo. The word lighter was added to the branding as a clear indicator of a choice with less calorific impact, without making claims about the relative healthiness of the products. The guidance used to categorize the products as healthy or unhealthy follows the same principle, making the calorific impact of choosing a product from this selection at the vending machine lighter than that of a standard product. Lighter choices was therefore deemed to be a good fit, particularly as simple, succinct health claims have been found to be more well-received than longer, more complex ones (Wansink, Sonka, & Hasler, 2004; Williams, 2005). The health advice labels therefore contained the words “Lighter choices”, and for the high source credibility manipulation, the NHS logo was added to the health advice label, to highlight the fact that the message was supported by a trusted, expert source.

SI.1. Planogram for vending machine products.

| #11  Walkers Baked Cheese + Onion | | #13  Walkers Baked Cheese + Onion | | #15  French Fries Ready Salted | | #17  French Fries Ready Salted | |
| --- | --- | --- | --- | --- | --- | --- | --- |
| #21 Walkers Baked Salt + Vinegar | | #23 Popchips BBQ | | #25 Popchips Sour Cream+ Onion | | #27 Popchips Sour Cream+ Onion | |
| #31 McCoys Flame Grilled Steak | | #33 McCoys Flame Grilled Steak | | #35 Hula Hoops BBQ | | #37 Hula Hoops BBQ | |
| #41 Quavers | | #43 Quavers | | #45 Tyrrell’s Roast Chicken | | #47 Tyrrell’s Sea Salt  & Cider Vinegar | |
| #50  Polo  S/F | #51  Nakd Cocoa Crunch | #52  Nakd Salted Caramel | #53  Nakd Blueberry Muffin | #54  Nakd Peanut Delight | #55  Nakd Cocoa & Orange | #56  Go Ahead Crispy Slices | #57  Go Ahead Crispy Slices |
| #60  KitKat | #61  KitKat | #62  Twix | #63  Twix | #64  Galaxy Smooth Milk | #65  Galaxy Caramel | #66  Caramel Flapjack | #67  Chocolate Flapjack |

Healthy rows in green, unhealthy in red. Numbers indicate positions within vending machine.

SI.2. Nutritional information for all products.

| **Product number** | **Product** | **Kcals** | **Fat**  **(g)** | **Saturated fat  (g)** | **Carbohydrate (g)** | **Sugars (g)** | **Sodium (g)** |  |  |  |  |  |  |
| --- | --- | --- | --- | --- | --- | --- | --- | --- | --- | --- | --- | --- | --- |
| 1 | French Fries Ready Salted | 91 | 3.4 | 0.30 | 13.7 | 0.2 | 0.53 |  |  |  |  |  |  |
| 2 | Go Ahead Crispy Slice Apple | 165 | 3.0 | 0.03 | 32.7 | 13.8 | 0.27 |  |  |  |  |  |  |
| 3 | Nakd Blueberry Muffin | 130 | 4.6 | 0.70 | 19.0 | 18.2 | 0.10 |  |  |  |  |  |  |
| 4 | Nakd Cocoa Orange | 145 | 7.0 | 1.50 | 15.8 | 13.6 | 0.10 |  |  |  |  |  |  |
| 5 | Nakd Cocoa Crunch | 105 | 2.6 | 0.60 | 14.2 | 12.9 | 0.20 |  |  |  |  |  |  |
| 6 | Nakd Peanut Delight | 149 | 7.5 | 1.40 | 14.6 | 13.6 | 0.20 |  |  |  |  |  |  |
| 7 | Nakd Salted Caramel | 124 | 4.6 | 0.80 | 18.6 | 17.7 | 0.30 |  |  |  |  |  |  |
| 8 | Polo Sugar Free | 78 | N/A | N/A | 32.9 | 0.1 | < 0.01 |  |  |  |  |  |  |
| 9 | Popchips BBQ | 97 | 3.6 | 0.30 | 14.0 | 2.1 | 0.49 |  |  |  |  |  |  |
| 10 | Popchips Sour Cream & Onion | 95 | 3.4 | 0.40 | 15.0 | 1.0 | 0.45 |  |  |  |  |  |  |
| 11 | Walkers Baked Cheese & Onion | 163 | 5.1 | 0.50 | 25.7 | 2.8 | 0.35 |  |  |  |  |  |  |
| 12 | Walkers Baked Salt & Vinegar | 162 | 5.0 | 0.50 | 25.5 | 2.4 | 0.30 |  |  |  |  |  |  |
| 13 | Caramel Flapjack | 442 | 23.0 | 7.80 | 55.0 | 31.0 | 0.60 |  |  |  |  |  |  |
| 14 | Chocolate Flapjack | 446 | 22.0 | 9.70 | 54.0 | 25.0 | 0.18 |  |  |  |  |  |  |
| 15 | Galaxy Caramel | 232 | 11.0 | 7.00 | 30.0 | 27.0 | 0.08 |  |  |  |  |  |  |
| 16 | Galaxy Smooth Milk | 229 | 14.0 | 8.00 | 23.0 | 23.0 | 0.05 |  |  |  |  |  |  |
| 17 | Hula Hoops BBQ | 172 | 8.8 | 0.90 | 21.0 | 0.5 | 0.80 |  |  |  |  |  |  |
| 18 | KitKat | 209 | 10.2 | 5.70 | 26.1 | 21.3 | 0.10 |  |  |  |  |  |  |
| 19 | McCoys Flame Grilled Steak | 250 | 15.0 | 1.30 | 25.0 | 1.1 | 0.71 |  |  |  |  |  |  |
| 20 | Quavers | 107 | 6.2 | 0.50 | 12.0 | 0.5 | 0.43 |  |  |  |  |  |  |
| 21 | Twix | 248 | 12.0 | 7.00 | 32.2 | 24.4 | 0.22 |  |  |  |  |  |  |
| 22 | Tyrrell’s Roast Chicken | 204 | 11.5 | 1.20 | 21.4 | 1.5 | 0.32 |  |  |  |  |  |  |
| 23 | Tyrrell’s Sea Salt & Cider Vinegar | 195 | 10.4 | 1.10 | 22.4 | 0.7 | 0.70 |  |  |  |  |  |  |
|  | | | | | | | |  |  |  |  |  |  |

SI.3. Labels

Low credibility:


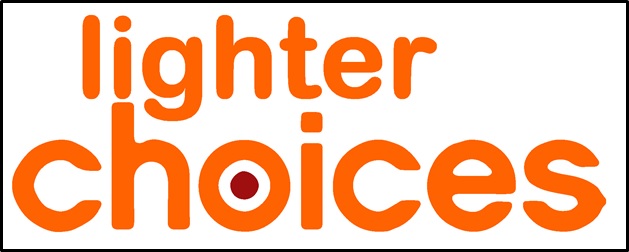


High credibility:


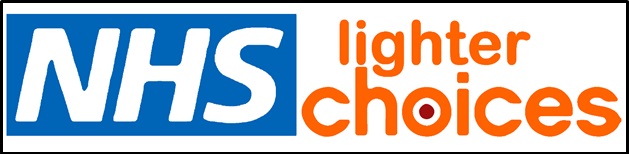


Low credibility label in situ:


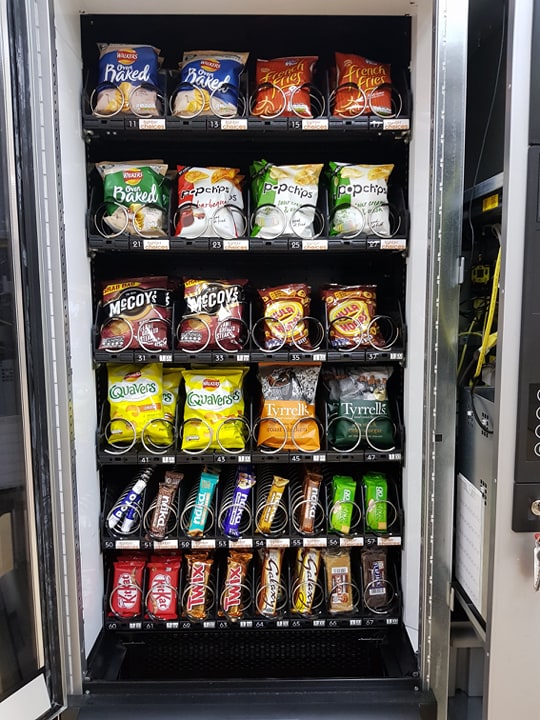


High credibility label in situ:


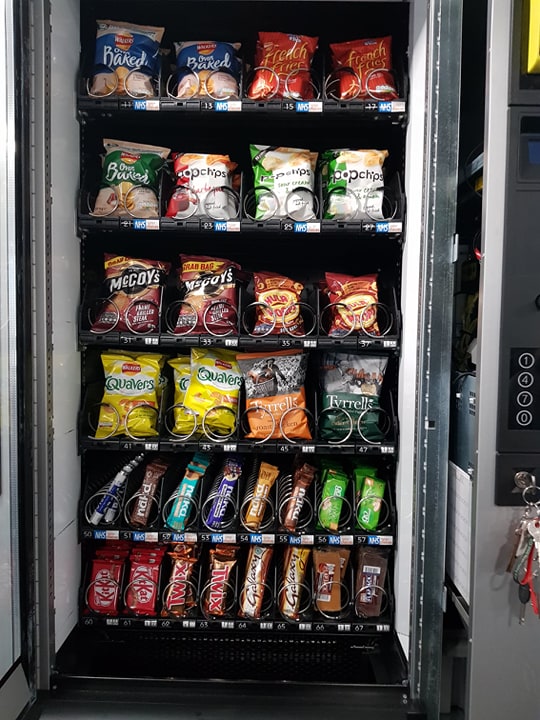

Supplement: S1 Methods — (DOCX) [file pone.0296901.s001.docx]
